# Retraction: Does Occupational Exposure to Solvents and Pesticides in Association with Glutathione S-Transferase A1, M1, P1, and T1 Polymorphisms Increase the Risk of Bladder Cancer? The Belgrade Case-Control Study

**DOI:** 10.1371/journal.pone.0349169

**Published:** 2026-05-12

**Authors:** The PLOS One Editors

After this article [1] was published, the Ministry of Health of Republic of Serbia and the Ethics Board of Serbia contacted PLOS about concerns with the ethics approvals in [1] and requested the retraction of [1]. Specifically, a representative of the Ethics Board of Serbia stated that the study in [1] was conducted without the necessary approval from the Ethics Board of the health institution (the University Clinical Center of Serbia) whose patients and biological material are the subject of the research in [1].

The first author stated that [1] was conducted with all the necessary approvals required at the time of sample collection and later data analysis, and provided copies of the ethics approval from the Ethics Committee of the Faculty of Medicine University of Belgrade and a letter of permission from the Director of the Clinic of Urology, University Clinical Center of Serbia.

A representative of the University Clinical Center of Serbia stated that the study in [1] was not reviewed and approved by the Ethics Board of the University Clinical Center of Serbia. They stated that the procedure for review and approval by the Ethics Committee of the Faculty of Medicine University of Belgrade and written consent from the Director of the Clinic of Urology, University Clinical Center of Serbia was in accordance with the Rulebook on Doctoral Studies of the University of Belgrade, which did not include specifics in the case of research involving patients.

PLOS did not receive a response from the University of Belgrade about the concerns raised for this study.

In light of concerns that the relevant approvals for clinical research involving patients were not in place at the time the study reported in [1] was conducted, and based on input from the Ethics Board of Serbia and the University Clinical Center of Serbia, the *PLOS One* Editors retract this article.

TDP agreed with the retraction. MGM, VMC, ARSR, PVB, MSPE, DPD, TID, and TPS did not agree with the retraction.

## References

[1] Matic MG, Coric VM, Savic-Radojevic AR, Bulat PV, Pljesa-Ercegovac MS, Dragicevic DP, et al. RETRACTED Does occupational exposure to solvents and pesticides in association with glutathione S-transferase A1, M1, P1, and T1 polymorphisms increase the risk of bladder cancer? The Belgrade case-control study. PLoS One. 2014;9(6):e99448. doi: 10.1371/journal.pone.0099448 24914957 PMC4051772

